# Active Learning with Bayesian UNet for Efficient Semantic Image Segmentation

**DOI:** 10.3390/jimaging7020037

**Published:** 2021-02-17

**Authors:** Isah Charles Saidu, Lehel Csató

**Affiliations:** 1 Department of Computer Science, African University of Science and Technology, Abuja, Nigeria; 2 Depterment of Mathematics and Informatics, Babeş–Bolyai University, RO-400084 Cluj-Napoca, Romania; lehel.csato@cs.ubbcluj.ro

**Keywords:** Bayesian, active learning, Bayesian learning, convolutional networks, AB-UNet, stochastic gradient descent

## Abstract

We present a sample-efficient image segmentation method using active learning, we call it Active Bayesian UNet, or AB-UNet. This is a convolutional neural network using batch normalization and max-pool dropout. The Bayesian setup is achieved by exploiting the probabilistic extension of the dropout mechanism, leading to the possibility to use the uncertainty inherently present in the system. We set up our experiments on various medical image datasets and highlight that with a smaller annotation effort our AB-UNet leads to stable training and better generalization. Added to this, we can efficiently choose from an unlabelled dataset.

## 1. Introduction

Semantic image segmentation—the task of clustering image pixels into categories—has been actively researched by the computer vision community [1]. In the last 50 years, these methods have included thresholding, pixel clustering, detection, watershed techniques, to name only a few [2,3,4,5,6,7,8]. With the new wave of neural networks, segmentation is done with variants thereof, either (1) pixel-wise or (2) superpixel classification. These recent—deep learning—methods led to improved performance in image classification and image segmentation [9,10,11,12,13,14]. As of today, most state-of-the-art segmentation rely on convolutional networks (CNN—a neural network with shared weights—the convolutions—that lead to translation invariance [15], and therefore good performance in image processing), outperforming methods that use low-level feature extraction [10,13,14]. We develop a neural network-based algorithm that addresses the difficulty of collecting the training data: to achieve segmentation, for each image we need pixel-wise labelling, that is extremely costly. We “informatively” sample from an un-labelled set of data and ask to label only the images that are “the most uncertain”. The result—the AB-UNet algorithm—achieves fast and accurate segmentation with a small set of annotated data.

### 1.1. Problem Statement and Suggested Solution—The AB-UNet Algorithm

We aim at an iterative segmentation with a minimal set of annotated images to minimise annotation effort. We leverage on the success of convolutional networks in image segmentation [13] and develop a probabilistic algorithm that uses active learning for training.

Our algorithm outputs pixel-wise uncertainty that is subsequently used for unlabelled image selection in inter-active training. For deep models, pixel-wise uncertain image selection is non trivial as it involves accounting for the noise in model as well as data, more so, each pixel uncertainty has to be summarized to estimate the informativeness of the image. We call our approach active Bayesian UNet—in abbreviated form AB-UNet. In the algorithm we “actively choose” the most informative image to be labelled. This process is done by a domain expert, usually called the “oracle” (which is a labelling agent—human or algorithmic—that performs the given task and providing the required output—image segmentation in this case)—leading to a model with the following properties:

- *Pixel-wise segmentation* of the image—no need for manual labelling of the training set.
- *Efficient and fast active training* via *informative scoring*, achieving good generalization. Also, ensure faster training after each interaction between model and the oracle.
- *Translation, rotation and scale invariance* to improve generalisation.

Our contribution is the Active Bayesian UNet algorithm. It is a classical UNet [13] with a Bayesian extension based on max-pool dropout [16,17,18], that uses batch normalization [19]. The probabilities quantize the uncertainty, leading to the possibility to choose informative samples. The informativeness of data is used in an “active learning” scenario—see Section 4—and we show that a committee-based Jensen divergence measure for the acquisition function (see Section 4.1—inspects the unlabelled set and returns “the most informative” item) achieves the best dice coefficient (Dice coefficient—see Section 3.3.2), and this value will certify the validity of our methods.

### 1.2. Structure of the Paper

In Section 1.3 we define notations, in Section 2 gives a brief discussion on related work. We begin the introduction of our extended UNet in Section 3. This section also includes brief definitions of Bayesian neural networks and Monte Carlo estimates with dropout and batch normalization. Subsequently, we present details of the AB-UNet architecture with the proposed Bayesian sampling technique. We justify this extension with empirical tests in Section 3.3.1. Section 4 introduces active learning and describes the proposed acquisition functions for selecting informative images. Further down, we present our AB-UNet algorithm. Section 4.4 presents empirical results of these acquisition functions, while Section 5 discusses conclusions and future work.

### 1.3. Notation and Assumptions

We denote $X_{j}$ as the jth image and $Y_{j}$ its corresponding segmentation mask in our dataset D. We assume that each $Y_{j}$ takes on pixel values in the range of 0,1,2,…,c−1, where *c* is the number of classes. In active learning, we have a small labeled dataset $X_{\ell }=\left\{X_{1},X_{2},\ldots ,X_{\ell }\right\}$ and $Y_{\ell }=\left\{Y_{1},Y_{2},\ldots ,Y_{\ell }\right\}$ such that the initial training set $D_{\ell ,1}=\left\{X_{\ell },Y_{\ell }\right\}$, with ℓ≪n and $X_{u}=X_{u}\backslash X_{\ell }$. We further assume the availability of a pool of unlabelled data $X_{u}=\left\{X_{1},X_{2},...,X_{n}\right\}$, and the existence of an oracle or a labeller who is an expert in the learning domain. The oracle is simulated by holding out a subset of already—ground truth labelled set $X_{u}$—and when selected—the “output” mask is made available.

## 2. Active Learning for Image Segmentation

Research into image segmentation spanning the last 50 years has seen groundbreaking results, where “classical” techniques, like thresholding, pixel clustering and edge detection [1] were the building blocks of the algorithms. In last years, there is a wave of algorithms using artificial neural networks (ANNs) and we mainly discuss algorithms that use ANNs. In spite of the large literature in segmentation, there are only a few that actively select data. Instead of selecting, the emphasis was on improving the accuracy of the segmentation task-as evident in research that uses deep neural networks like UNet, FCN [13,14] and Masked R-CNN [12]. In this article, we focus on these active learning literatures and further categorize them into two parts: (1) Graphical model-based approaches, and (2) Neural network-based approaches.

*1—Graphical model-based* semantic segmentation with active learning is one of the early techniques to build segmentation models that simultaneously aim to minimize annotation efforts. This technique constructs graphs where nodes are super-pixels (part of an image that is rendered with “almost” uniform colour and brightness) and edges are similarities between super-pixels [5,20,21].

Vezhnevets et al. [20] uses the graph based method with conditional random fields (CRFs) over super-pixels; the goal is to define an energy function that captures both the ability to classify super-pixels (unary potential) and the connectedness of super-pixels. In their work, they applied active learning by designing a query scoring function that maximizes the expected model change on the appearance model parameters.

Fathi et al. [21] focused on semantic video segmentation by building a graph of super-pixels connected via a similarity metric. Here an incremental self-training approach was proposed that iteratively first labels the least uncertain frame, followed by the update of similarity metrics based on the extended set of labels.

*2—Among neural-network based* approaches there are a few that address active learning aimed at image segmentation. The few works that exists exploit model uncertainty and pixel information evaluated using different flavours of entropy of posterior predictive pixel distribution.

Kendall and Gal [22] proposed a Bayesian network with heteroscedastic uncertainty that combines input-dependent uncertainty—coming from observation noise—with epistemic uncertainty—from the model—, resulting in a predictive pixel distribution with pixel information evaluated using entropy. Their Bayesian network is realized using dropouts with a special parameter regularization term (see [22] for details).

Mahapatra et al. [23] employs the gains inherent in deep neural networks by proposing an active learning technique and selection sampling technique using conditional generative adversarial networks (cGANs). Their framework has three components: (1) sample generation; (2) classification/segmentation model; (3) sample informativeness calculation. Uncertainty of samples is evaluated using a Bayesian neural network with heteroscedastic uncertainty [22] and informativeness of samples is evaluated using a summary of pixel entropies.

Gorriz et al. [24] proposed a closely related work in which they applied standard dropout on UNet architecture and a Monte Carlo average of weighted pixel prediction from the final/last layer of the network. Uncertainty in predicted samples is evaluated using maximum variance of *T* forward passes and the informativeness of each image is a summary of pixel entropies.

Our previous work [25] employs a superpixel-classification approach for prostate segmentation. We considered a training pipeline which started by weakly segmenting and over-sampling (using SMOTE sampling [26]) the input images, starting from a watershed algorithm—outputting several super-pixels. These super-pixels were the basis for object detection and it was shown that Bayesian Active learning by disagreement [27] acquisition functions outperformed other acquisition methods benchmarked.

## 3. The Extended UNet Architecture—The AB-UNet

In what follows we describe the standard UNet and the extension to allow the active learning within the segmentation task.

### 3.1. Standard UNet Convolutional Network

**Figure 1:**
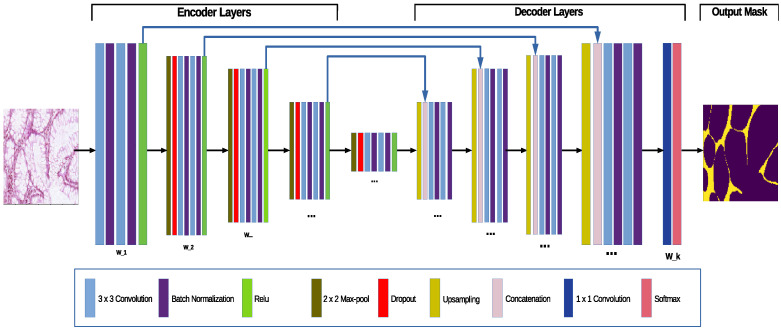
The Bayesian UNet—an extension of standard UNet—where batch normalization and max-pool dropout for posterior weight sampling were added to the architecture.

The standard UNet proposed by Ronneberger et al. [13] consists of a contractive (encoder) convolutional part and an expansive (decoder) convolutional part, forming a U-shape, hence its name. The contractive part consists of a rectifier unit (A rectifier is an activation function defined as the positive part of its argument f(x)=max(0,x)) placed after every second convolutional layer, the result is then downscaled using a max pool layer. This contraction reduces the spatial information, while increasing feature information [13]. The expansive pathway combines the feature and spatial information through a sequence of up-convolutions and concatenations with high-resolution features from the contracting path (see Figure 1). In what follows we describe the probabilistic extension of the classical UNet algorithm.

### 3.2. Bayesian Neural Networks

A Bayesian neural network is one with a probability distribution over its network weights; an immediate advantage being the fully probabilistic treatment, hence the estimation of the uncertainties in predictions. In a Bayesian setting, we assume a prior knowledge or distribution $p_{0}\left(W\right)$ of these weights and estimate the posterior weights distribution p(W|D) after observing the data D. This posterior weight distribution is evaluated using Bayes rule as

(1) $$p\left(W|D\right)=\frac{p\left(D|W\right)p_{0}\left(W\right)}{p(D)}$$

where $p_{0}\left(W\right)$ is the a-priori weight distribution—usually an isotropic Gaussian—and p(D) is the normalizing constant of the distribution. Under this setting, predictions are done using the posterior from Equation (1):

(2) $$p\left(Y^{*}|X^{*},D\right)=\int p\left(Y^{*}|,X^{*},W\right)p\left(W|D\right)dW$$

In our work the uncertainties arise due to sampling the dropout weights and batch normalization, and prediction is done by averaging *T* forwarded passes over the network (MCMC procedure):

(3) $$p\left(Y^{*}|X^{*},D\right)=\frac{1}{T}\sum_{t=1}^{T}p\left(Y^{*}|X^{*},D,W_{t}\right)$$

In what follows we present the network layers (maxpool, batch normalization) and their respective contributions to uncertainty estimation, as seen in Figure 1:

1 Batch Normalization [19] is a procedure to speed up network training by reducing the internal covariate shift (this describes the changes in the distributions of activation units due to changes in parameters [19]) done by normalizing the hidden layers activations using an estimated $\mu_{\beta }$ and $\sigma_{\beta }$ from each mini-batch. Teye et al. [18] found that batch normalization helps improving convergence.
2 Dropout [16] is a regularization technique, also viewed as an approximate Bayesian method: the algorithm randomly removes parts of the network, making the weights stochastic quantities: $\hat{W}=W⊗\alpha$, where α∼Bernoulli(p), W are the initial weights of the network, and ⊗ is the direct product with the random binary vector.

The network is trained using the dropout $\hat{W}$, the training method is stochastic gradient descent, leading to both uncertainty and robustness.

### 3.3. The Probabilistic Extension, the AB-UNet Architecture

We present our Bayesian UNet (AB-UNet)-an extension of the standard architecture via sampling. We place batch normalization layer after two consecutive convolutional layer and dropout after each max pool layer—the architecture is shown in Figure 1. The AB-UNet contains stochastic parameters $\Theta =\{{\hat{W}}^{1..L},\mu_{\beta }^{1..L},\sigma_{\beta }^{i..L}\}$ where *L* is the number of layers in the network. Similar to the standard UNet, we define the softmax output vector $p{\left(y=i|x,D,\Theta \right)}_{i=1}^{c}$ with *c* being the number of pixel classes, and a categorical cross-entropy loss for our model. The network is subsequently trained using stochastic gradient descent with Adam [28]. The prediction is done by averaging forward passes as in Equation (3).

Recent researches have shown that convolutional layer dropouts are hard to train, can lead to unstable behaviour [29] with high uncertainty in predictions. We resolved this issue by carefully placing dropouts at different layers of the network and empirically benchmarking the results. The result of this experiment led to placing dropouts after each max pool hence forming the basis of our AB-UNet architecture and active learning algorithm. We present the experiment as follows.

#### 3.3.1. Model Performance and Uncertainty Quantification AB-UNet

We setup various experiments to compare dropout performance/uncertainty estimates together with batch norm performance/uncertainty estimates across various datasets. Beginning with a very small training set size to a large training set sizes, we investigated the performance and uncertainty estimate using dice coefficient (see Section 3.3.2) of our segmentation. The goal is to find the best performing model with the smallest dataset size, while guaranteeing also stability.

#### 3.3.2. Dice Coefficient

Also known as the Sorensen Index or F1 score—developed independently by Lee R. Dice and (Thorvald) Julius Sorensen in 1945 and 1948 respectively [30,31], this is a metric that computes similarity between two samples/vectors by balancing the trade-off between their precision (=true positive/(true positive + false positive)) and recall (=true positive/(true positive + false negative)). It is defined as $DSC=\frac{2||a⊙b||}{{\left||a|\right|}^{2}+{\left||b|\right|}^{2}}$, where ⊙ is the element-wise multiplication, and a,b are vectors.

In our work, we use the dice coefficient to measure the similarity between predicted and ground truth segmentation.

#### 3.3.3. The Results of Model Uncertainty Quantification

**Figure 2:**
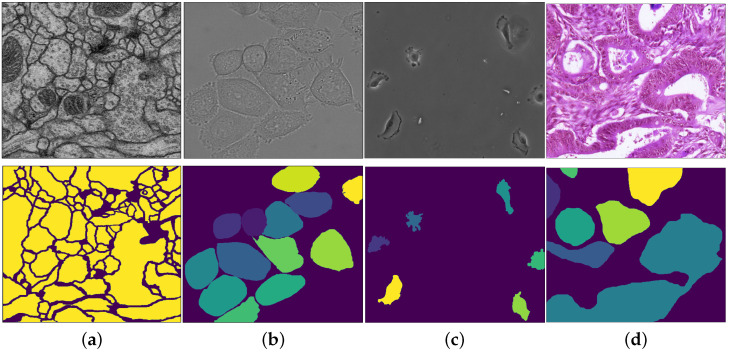
(top) Images used in the experiments and (bottom) their corresponding segmentation masks: (**a**) membrane dataset (2 classes), (**b**) differential interference contrast (DIC)-C2DH-Hela dataset (20 classes), (**c**) PhC-C2DH-U373 dataset (14 classes), (**d**) Qu-Warwick dataset (50 classes).

In the experiments we used batch normalization and we benchmarked the following four versions of our algorithm: (1) plain, (2) standard dropout, (3) max-pool dropout, and (4) both standard and max-pool dropouts. The benchmarking is on four datasets—see Figure 2 for typical images:

1. *Cell membrane segmentation dataset* [32] from the EM segmentation challenge. It contains a full stack of EM slices images used to train machine learning models for automatic segmentation of neural structures. These images contain noise and small image alignment errors. For our experiments we discretized each pixels as binary values.
2. *DIC-C2DH-HeLa* (The dataset is provided by Dr. Gert van Cappellen, from the Erasmus Medical Center, Rotterdam, The Netherlands.) cell tracking dataset of images recorded by differential interference contrast (DIC) microscopy. We discretized each pixel in this dataset into 20 classes.
3. *PhC-C2DH-U373 dataset*:*The data is provided by Dr. Sanjay Kumar. Department of Bio-engineering University of California at Berkeley. Berkeley CA (USA).* on Glioblastoma-astrocytoma U373 cells on a polyacrylimide substrate recorded by phase contrast microscopy. For our segmentation experiments we used 14 classes.
4. Warwick gland segmentation in colon histology images dataset [33]. This dataset consists of images of Hematoxylin and Eosin (H&E) stained slides, consisting of a variety of histologic grades (Figure 2). The dataset is provided together with ground truth annotations by expert pathologists and the task is to build an algorithm that segments the glands within the image. For our experiment we discretized each pixel into 50 classes.

**Figure 3:**
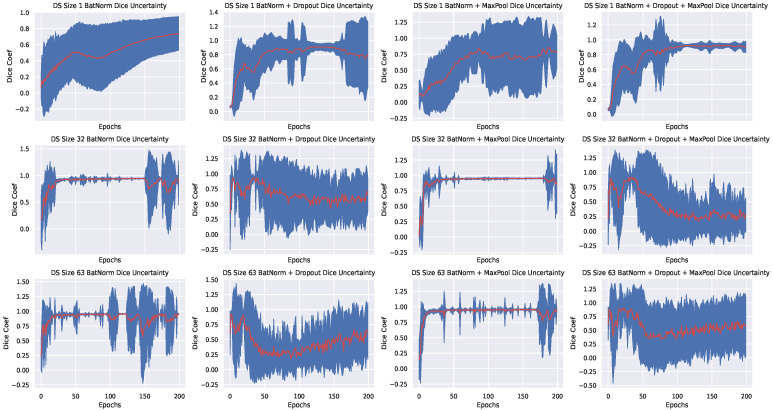
Validation DICE coefficients and their uncertainty for the PhC-C2DH-U373 dataset using different Bayesian approximations, where shading indicates the predictive variance. Figures are arranged based on the different type of experiments carried across different training set sizes: 1,32,63.

We augmented (rotated, shifted, scaled and sheared) each dataset to improve generalization. We ran each experiment—a permutation of plain, standard dropout, max-pool dropout and both, for a total of 10 times each at 200 epochs. Training dice coefficient, validation dice coefficient, training loss, validation loss, Markov Chain Monte Carlo (MCMC) validation dice coefficient and Markov Chain Monte Carlo (MCMC) validation loss results were averaged, plotted and the following were observed:

1. By using batch normalization and max-pool dropout, we achieved better generalization and uncertainty quantification on all datasets; in contrast to batch normalization + standard dropout only, or batch normalization + standard dropout + max-pool dropout. Using only Batch normalization, exhibited similar model confidence when compared with batch normalization and max-pool dropout but it resulted to a slightly lower dice coefficient values across various sizes of training dataset.
2. Better uncertainty (We define better uncertainty as the confidence of the model when it has seen more data) with more data: this is observed via the low variance in the plots shown in Figure 3—for the PhC-C2DH-U373 dataset, but the training behaviour for other datasets is similar. We see that our Bayesian model trained with either batch normalization + max-pool dropout or batch normalization only exhibit better confidence as the size of the training set increases—a clear contrast with the other setups. In particular, model confidence is better exhibited when using batch normalization and max-pool dropout.
3. Average generalization begins below 60 epochs-An observation that we later exploited in active learning retraining (see Section 4.4). We believe that the fast generalization is a result of the batch normalization of input features. This is because batch normalization has been shown to reduce internal covariant shift, resulting to faster training and convergence [19].

## 4. Active Learning—A More Data-Efficient Method

Active learning is a sub-field of machine learning that holds the hypothesis that a learning algorithm can achieve greater accuracy with fewer labelled samples if it is allowed to interactively select its own training data points and request their corresponding labels from an oracle. Classical active learning can be categorized into pool-based (Pool-based active learning assumes a pre-defined and available unlabelled data, usually of fixed size) active learning, stream-based (In stream-based active learning we assume that data arrives in streams—online setting—and the model decides whether or not to query its label) active learning, and membership query synthesis (In membership query synthesis we assume that unlabelled data is synthesized from labelled ones) see [34] for a detailed description.

An essential part of active learning is the acquisition function that determines which item from the unlabelled data points is selected for in-depth labelling. Depending on the model, the acquisition function exploits the uncertainty in models (for probabilistic models) or the distance between data points and a separating hyperplane (for non-probabilistic models).

In AB-UNet, choosing informative images is peculiar since for each predicted pixel there is a distribution, therefore, we are faced with the problem of measuring the uncertainty of not just the pixel prediction but the entire input image. We denote the informativeness of an image as $I(X_{j})$ and propose two categorizes of acquisition functions: (1) entropy-based techniques, (2) divergence based techniques-committee based techniques.

### 4.1. Acquisition Functions for Active Learning

1. Entropy based techniques compute the informativeness of an image as the sum of pixel entropies within the image. We define the following cases:
  (a) Maximum entropy [35]: measures the informativeness of pixel predictions within the image. The entropy of a pixel $x_{i}$ is $\hbar_{e}\left(x_{i}\right)=H_{x_{i}\in X_{j}}\left[y|x_{i}\right]$. Therefore, $I\left(X_{j}\right)=\sum_{x_{i}\in X_{j}}\hbar_{e}\left(x_{i}\right)$
  (b) BALD (Bayesian Active learning by disagreement) [27]: chooses the image that maximizes the mutual information between the standard prediction and posterior prediction of each pixel. The BALD of a pixel $x_{i}$ is thus defined as $\hbar_{b}\left(x_{i}\right)=H\left[y|x_{i}\right]-E_{p(\Theta |D)}\left[y|x_{i}\right]$. Therefore, $I\left(X_{j}\right)=\sum_{x_{i}\in X_{j}}\hbar_{b}\left(x_{i}\right)$
2. Divergence based techniques: Computes the divergence between standard model prediction and MCMC prediction, therefore taking into account the disagreements of predictions in weight space while also considering noise in data space. We consider the following variants:
  (a) Committee posterior KL-divergence: computes the divergence between standard predictions and posterior predictions: given p(Y|X,Θ), the prediction from our AB-UNet model, and $E_{\Theta }\left(Y|X,\Theta \right)$ our MCMC prediction, we define the $D_{KL}\left(p\left(Y|X,\Theta \right)||E_{\Theta }\left(Y|X,\Theta \right)\right)$ as the information gained if we approximate p(Y|X,Θ) with our MCMC prediction $E_{\Theta }\left(Y|X,\Theta \right)$. Using this acquisition function, we select samples with the highest KL divergence.
  (b) Committee posterior Jensen divergence is similar to the KL divergence, but here we quantize the symmetric bi-directional divergence between the standard prediction and the MCMC predictions. The Jensen divergence is defined as $\mathrm{JSD}\left(p||Q\right)=\frac{1}{2}D_{kl}\left(p||M\right)+\frac{1}{2}D_{kl}\left(Q||M\right)$ where $M=\frac{1}{2}\left(p+Q\right)$, $p=p\left(Y|X,\Theta \right)–\mathrm{standard}\;\mathrm{prediction},\;\mathrm{and}\;Q=E_{\Theta }\left(Y|X,\Theta \right)$–MCMC prediction.

### 4.2. The AB-UNet Algorithm

Our AB-UNet algorithm extends standard active learning algorithm by introducing acquisition functions suitable for our Bayesian UNet. The algorithm starts with a small set of labelled examples (with 2 labelled items). Subsequently we retrain our model with additional samples selected using the acquisition functions defined in Section 4.1, where a simulated oracle provides the labels—we simulate the oracle providing labels by holding out labels for the unlabelled dataset and providing it when requested by our algorithm. At each interaction step(model and oracle), we refine the trained weights from previous iterations, as opposed to the re-initialization of the weights at each iteration. All training is done using Adam [28] optimizer with a learning rate of 0.001. In Section 3.3.3 we established that generalization occurs below 60 epochs so we employ early stopping technique with validation dice coefficient metric as stopping criteria—this generally speeds-up retraining. The algorithm is given in Algorithm 1. We emphasise that this is an inter-active training technique hence we expect the oracle to be present during training. Therefore, our focus is on avoiding to label the entire dataset. In practice, the oracle only needs to provide labels for the test set and the initial small training set; subsequent labels are only provided by the oracle on request by the algorithm.
**Algorithm 1:** The Active Convolutional Network Segmentation (Bayesian UNet) Algorithm1:**procedure**Training($X_{u}$)2:    Select $X_{init}$; $X_{u}\leftarrow X_{u}\backslash X_{init}$▹ Set of images from unlabelled set $X_{u}$3:    $Y_{init}\leftarrow \mathrm{oracle}\left(X_{init}\right)$▹ acquire label from Oracle4:    $D_{\ell ,1}\leftarrow \left(X_{init},Y_{init}\right)$5:    t←16:    $f_{t}\leftarrow \mathrm{Bayesian}\;\mathrm{UNet}\left(D_{\ell ,1}\right)$▹ initial training7:    **repeat**8:        $X_{sub}\subset X_{u}$9:        $S_{sub}\leftarrow \mathrm{unlabeledSet}\left(f_{t},X_{sub}\right)$▹ Computing score of $X_{sub}$10:        $X_{k}\leftarrow {arg max}_{X_{j}\in X_{u}}I\left(X_{j}\right)$▹ acquisition function $I(X_{j})$ from Section 4.111:        $Y_{k}\leftarrow \mathrm{oracle}\left(X_{k}\right)$▹ request labels12:        $D_{\ell ,t}\to D_{\ell }\cup \left(X_{k},Y_{k}\right)$13:        $X_{u}\to X_{u}\backslash X_{k}$14:        $f_{t+1}$$\leftarrow \mathrm{Bayesian}\;\mathrm{UNet}(D_{\ell ,t})$▹ re-train until early stopping15:        t←t+116:    **until**
$\mathrm{stopCondition}\vee X_{u}=Ø$17:    **return** trainedModel18:**end procedure**

### 4.3. Active Bayesian UNet Experiments

In our setup, we assume pool-based active learning with fixed size dataset and we select the unlabelled data points (images) from this set. We run our experiments on the four datasets, comparing all acquisition functions on each dataset. A total of 5 runs per experiment was done and the results of each MCMC validation DICE were averaged.

### 4.4. AB-UNet Algorithm Results

**Figure 4:**
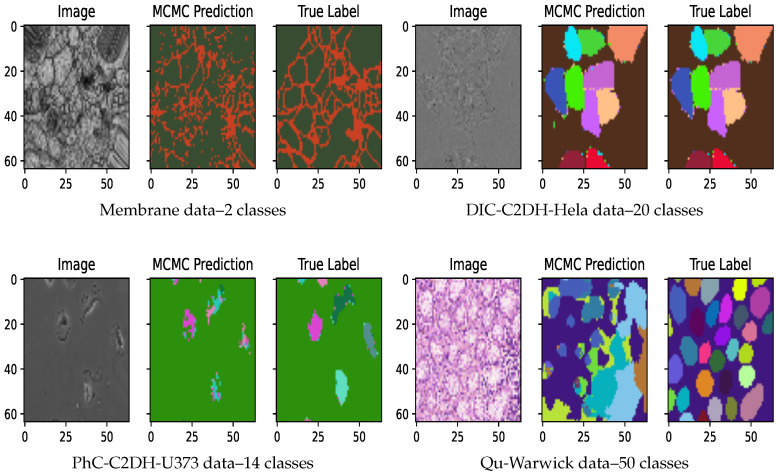
Predictions after 15 active learning iterations.

**Figure 5:**
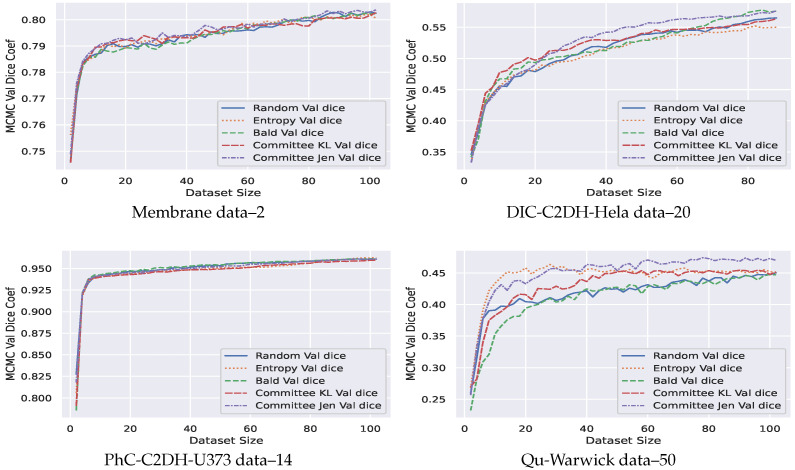
Markov Chain Monte Carlo (MCMC) validation DICE coefficient for active learning (datasets below images).

**Figure 6:**
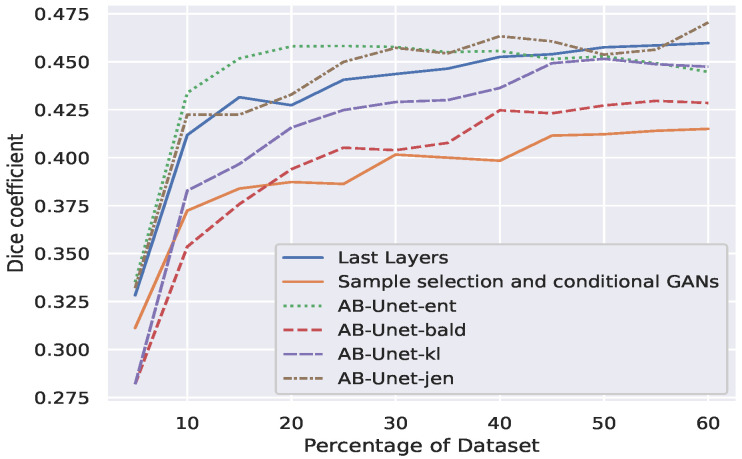
Comparative MCMC DICE coefficient results for Active Bayesian (AB)-UNet (label: AB-Unet-type) versus cost-effective active learning (label: Last layers) of Gorriz et al. [24], and Sample Selection and conditional generative adversarial networks (GANs) from Mahapatra et al. [23].

Our experiments show both marginal and significant improvements using AB-UNet in all datasets. Visual evidence is seen after 15 active learning iterations (using committee Jensen acquisition) with 2 most informative active batch samples added to training dataset at each iteration (see Figure 4). Overall, we observed the following:

- Our AB-Net shows significant early peak using entropy. However, a robust performance in terms of dice coefficient is observed for Jensen divergence acquisition function—a divergence based approach (Figure 5).
- Our technique is more effective for problems involving higher number of pixel classes. This is clearly seen in Figure 5: the Qu-warwick datasets has 50 classes, DIC C2DH Hela dataset has 20, PhC-C2DH-U373 dataset has 14 and Membrane dataset is binary. Comparing the performance of all datasets, we observe that the sample complexity for models trained using active learning is a function of the dimension of the classes.
- Finally we compared our AB-UNet technique with other related techniques in literature (see Section 2), using Qu-warwick dataset and the result is presented Figure 6. Observe that our AB-UNet outperforms these other techniques by a good margin and the committee-Jensen acquisition is comparatively better than entropy and KL divergence, as more labels are acquired. In general our technique performed better due to the following;
  1. Our AB-UNet assumes that all layers are equality informative in calculating the uncertainty in prediction, this is in contrast to [24] that only samples the last layer for MCMC prediction. Results from our comparative analysis in Figure 6. justifies this assumption.
  2. The max-pool dropout and batch normalization act as regularizers in our model compared to the work by Mahapatra et al. [23].
  3. Our AB-UNet algorithm-with committee Jensen, better models differences in predictive distributions induced by weight-space as well as noisy data. This is in contrast to standard entropy used in [23], hence the stability of our method.
  4. The averaging term $M=\frac{1}{2}\left(P+Q\right)$ in the Jensen divergence, makes the resulting measure smooth, more robust and well defined, implying that its range is well quantized and suitable when used to quantify the informativeness of an image among other images.

## 5. Conclusions and Future Work

We presented AB-UNet: a sample-efficient segmentation method using active learning model. AB-UNet is a convolutional neural network whose Bayesian treatment is via batch normalization and max-pool dropout–a choice of which was motivated by empirical model comparison results. The uncertainty quantification experiments showed that AB-UNet trained with batch normalization and max-pool uncertainty achieves better dice coefficient on validation set and are more confident as the dataset size increases. This is a property we desired and exploited in algorithm design to improve label complexity and reduce annotation effort.

In the active learning experiments, we showed that by using committee Jensen divergence acquisition function, we achieve better performance in terms of dice coefficient. This function penalizes the divergence between standard prediction and the MCMC prediction of our model via active retraining. Therefore, by using committee Jensen divergence acquisition function, we achieve training with fewer request for labels while maintaining better generalization. Also, each predictive mask comes with uncertainty information, so in practice annotators can only focus on regions within the image that are most uncertain and provide the labels for those regions only.

In general, our technique is easy to implement, tractable and achieves faster generalization compared to other techniques in literature; with tractability achieved through early stopping technique, iterative weight tuning, effectively leading to a quickly trained model at each active learning iteration. We also note the slight overhead in MCMC predictions since we need to average *T* forward passes for each MCMC prediction, however, the prediction tasks can easily be parallelized.

Lastly we showed, empirically, that sample complexity of our active learning technique is a function of the number of pixel classes. Intuitively, higher number of classes, translates to higher uncertainty in the system, hence better information gain can be achieved via active learning.

In the future, we plan to exploit ratios of combination of informative and less informative samples so as to prevent a possibility of getting stuck in a local minima. As a step to further improving annotation cost, we shall be exploring the game theoretic approach of exploring/exploiting predictions from AB-UNet instead of directly requesting labels from oracle. 

## Data Availability

Data and codes available at https://github.com/charlesity/U_Net_experiments.
